# Nanoscale Dynamics of Protein Assembly Networks in Supersaturated Solutions

**DOI:** 10.1038/s41598-017-14022-7

**Published:** 2017-11-01

**Authors:** Y. Matsushita, H. Sekiguchi, C. Jae Wong, M. Nishijima, K. Ikezaki, D. Hamada, Y. Goto, Y. C. Sasaki

**Affiliations:** 1 Graduate School of Frontier Sciences, The University of Tokyo, 5-1-5 Kashiwanoha, Kashiwa, Chiba, Japan; 2 Japan Synchrotron Radiation Research Institute, SPring-8, 1-1-1 Kouto, Sayo, Hyogo, Japan; 3 Office for University - Industry Collaboration, Osaka University, 2-8, Yamadaoka, Suita, Osaka, Japan; 4 Graduate School of Engineering, Kobe University, 7-1-48 Minato-jima, Minami, Kobe, Hyogo, Japan; 5 SPring-8/RIKEN, 1-1-1 Kouto, Sayo, Hyogo, Japan; 6 Institute for Protein Research, Osaka University, 3-2 Yamadaoka, Suita, Osaka, Japan; 7 AIST-UTokyo Advanced Operando-Measurement Technology Open Innovation Laboratory (OPERANDO-OIL), National Institute of Advanced Industrial Science and Technology (AIST), Chiba, 277-8568 Japan

## Abstract

Proteins in solution are conventionally considered macromolecules. Dynamic microscopic structures in supersaturated protein solutions have received increasing attention in the study of protein crystallisation and the formation of misfolded aggregates. Here, we present a method for observing rotational dynamic structures that can detect the interaction of nanoscale lysozyme protein networks via diffracted X-ray tracking (DXT). Our DXT analysis demonstrated that the rearrangement behaviours of lysozyme networks or clusters, which are driven by local density and concentration fluctuations, generate force fields on the femtonewton to attonewton (fN – aN) scale. This quantitative parameter was previously observed in our experiments on supersaturated inorganic solutions. This commonality provides a way to clarify the solution structures of a variety of supersaturated solutions as well as to control nucleation and crystallisation in supersaturated solutions.

## Introduction

The microscopic behaviour of proteins in solution has received considerable attention in the study of protein crystallisation^1^. Protein crystallisation is a complex process because numerous factors, including structure size, charge, conformation and dynamic properties, affect nucleation and crystal growth^2^. To clarify the behaviour of proteins in supersaturated conditions, it is necessary to understand the crystallisation process, particularly during the early nucleation stage. This process plays important roles in protein biomineralisation, which controls morphology in natural systems^3^, as well as in protein-protein interactions and in abnormal protein aggregation, a phenomenon observed in several neurological disorders^4^.

Small-angle X-ray scattering^5,6^, small-angle neutron scattering^7–9^ and dynamic light scattering^10–12^ have made vast contributions to characterising the behaviour of protein molecules in supersaturated conditions. Recently, equilibrium protein clusters or networks in the range of several tens to hundreds of nanometres have attracted attention for their behaviour in highly concentrated or supersaturated solutions^8,13^.

Previously, our group reported a local dynamic analysis method for inorganic (i.e., sodium acetate) supersaturated solutions using the free-standing diffracted X-ray tracking (DXT) method^14^. In a previous study, we demonstrated that localised molecular networks generated an extremely small force field derived from the rearrangement or relaxation of molecular networks under dense molecular packing conditions. In this report, we used DXT to observe the local dynamic structures of protein networks in supersaturated solutions.

**Figure 1:**
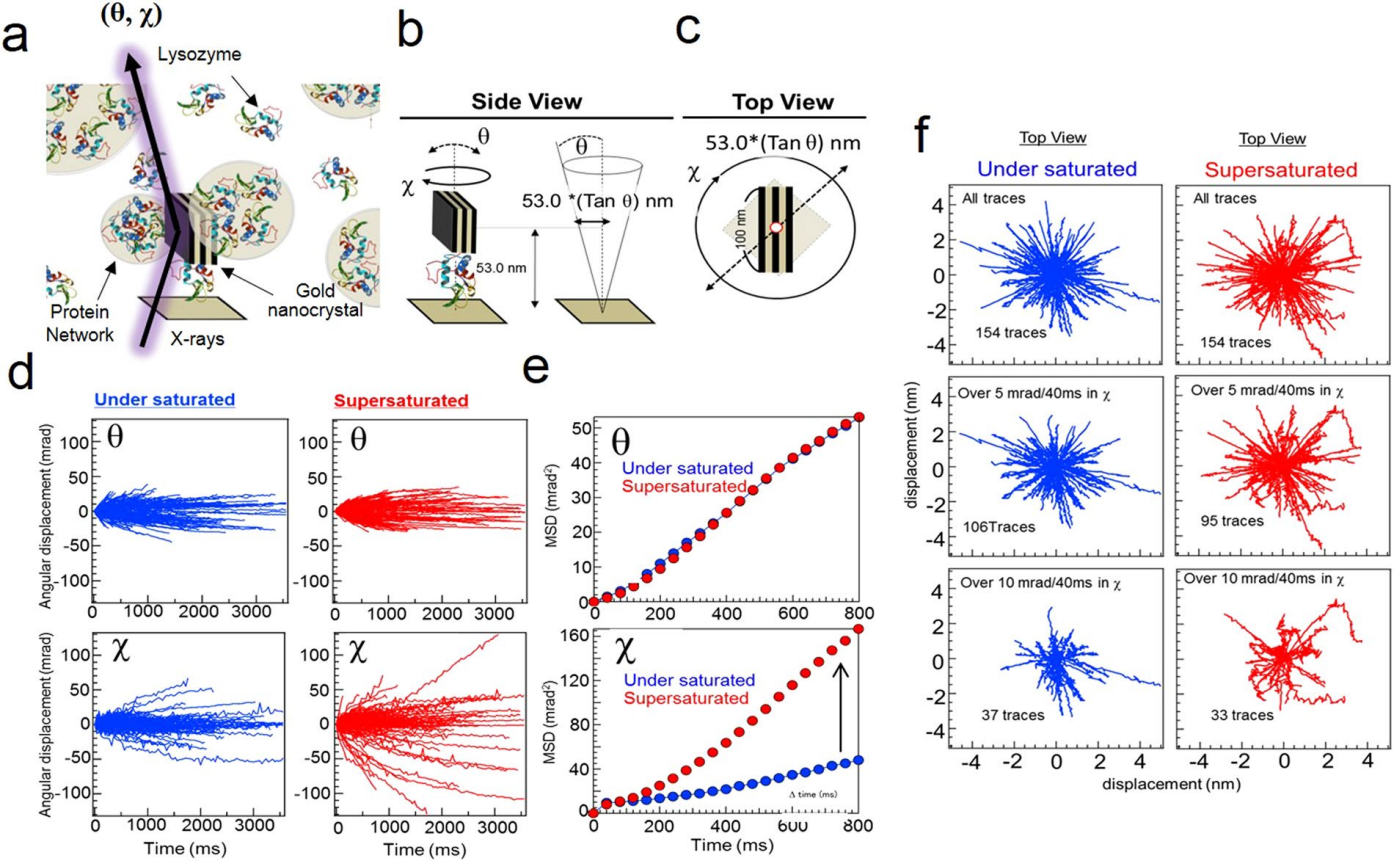
Statistical analysis of the rotational displacement of gold nanocrystals fixed on a substrate under saturated and supersaturated conditions. (**a**,**b**,**c**) Sample arrangement of the DXT measurement for observing the local dynamic structure under supersaturated conditions. An individual gold nanocrystal was fixed on the substrate via a lysozyme molecule. Rotational axes θ and χ were defined as shown in the figure. (**d**) Rotational angular displacement in the θ and χ directions for saturated and supersaturated lysozyme solutions. (**e**) Mean square displacement under saturated and supersaturated conditions. The supersaturated solution exhibited increased dynamics in the χ direction. f Transitional trajectories for an individual gold nanocrystal under saturated and supersaturated conditions. A removal process was performed for traces with velocities over 5 mrad/40 ms or 10 mrad/40 ms in the middle and the bottom, respectively.

DXT is an X-ray-based method for observing single molecules with high time resolution (μs) and high positional accuracy (pm)^14–16^. As shown in Fig. 1a–c, DXT can detect the rotational angular displacement of a gold nanocrystal bound on a substrate via a lysozyme molecule under supersaturated conditions. Rotational angular displacement of gold nanocrystals was observed in both the θ and χ directions (Fig. 1b). This sample arrangement was used because DXT is a specialised method for detecting small dynamic angular displacements (θ: −28 mrad) with highly accurate spatial resolution. As previously reported, the viscosity of a lysozyme solution (5–30 mg/mL solution: 1.28 · 10^−3^ Pa · s)^17^ is approximately three times lower than the viscosity of an inorganic supersaturated solution (3.18 · 10^−3^ Pa · s)^14^. For this reason, we tried to artificially limit gold nanocrystal dynamics in low-viscosity solutions to observe the dynamic properties of the lysozyme. Moreover, our previous research aimed to observe anisotropic force fields in supersaturated conditions via their effects on gold nanocrystal dynamics. The fixed gold nanocrystal condition shown in Fig. 1b was considered an adequate sample arrangement for calculating the anisotropic force field. Because the centre of the rotating gold nanocrystals remains static, translational motion of the gold nanocrystals can be avoided. Finally, the thiol functional groups in lysozyme molecules have a strong affinity for gold particles^18^, which allows for the incorporation of gold nanocrystals into the protein networks (Fig. 1a).

In this study, we characterised the behaviour of this protein in a supersaturated solution (16 mg/mL in 0.6 M sodium acetate with 9% w/v NaCl, pH 4.7) and in a saturated solution (5 mg/mL lysozyme in 0.2 M sodium acetate with 3% w/v NaCl, pH 4.7). The supersaturated conditions were confirmed via a UV-absorbance check between 200 and 400 nm that showed no amorphous aggregation (Figure S1); sea-urchin-shaped lysozyme crystals were formed by incubating the solution for approximately 6–12 hr at 25 °C (Figure S2).

Figure 1d shows the two-axis (θ and χ) rotational displacement of gold nanocrystals in supersaturated and saturated solutions over 3.6 seconds at a frame rate of 40 ms. The results showed no notable difference in gold nanocrystal dynamics under supersaturated and saturated conditions in the θ direction. By contrast, several traces under supersaturated conditions indicated a remarkable difference in the χ direction compared with the traces under saturated conditions. The mean square displacement (MSD) of rotational motion in the θ and χ directions revealed this trend, as shown in Fig. 1e. Figure 1f demonstrates that the tilting motion of the gold nanocrystals viewed from the top reached maximum values of approximately 3-4 nm during the 3.6 s duration of the experiment. Moreover, we filtered the traces and selected only those with rotational angle displacements that were larger than either 5 mrad or 10 mrad for 40 ms in the χ direction. This filtering step identified several traces in which the gold nanocrystals changed their rotational direction during measurement under supersaturated conditions.

**Figure 2:**
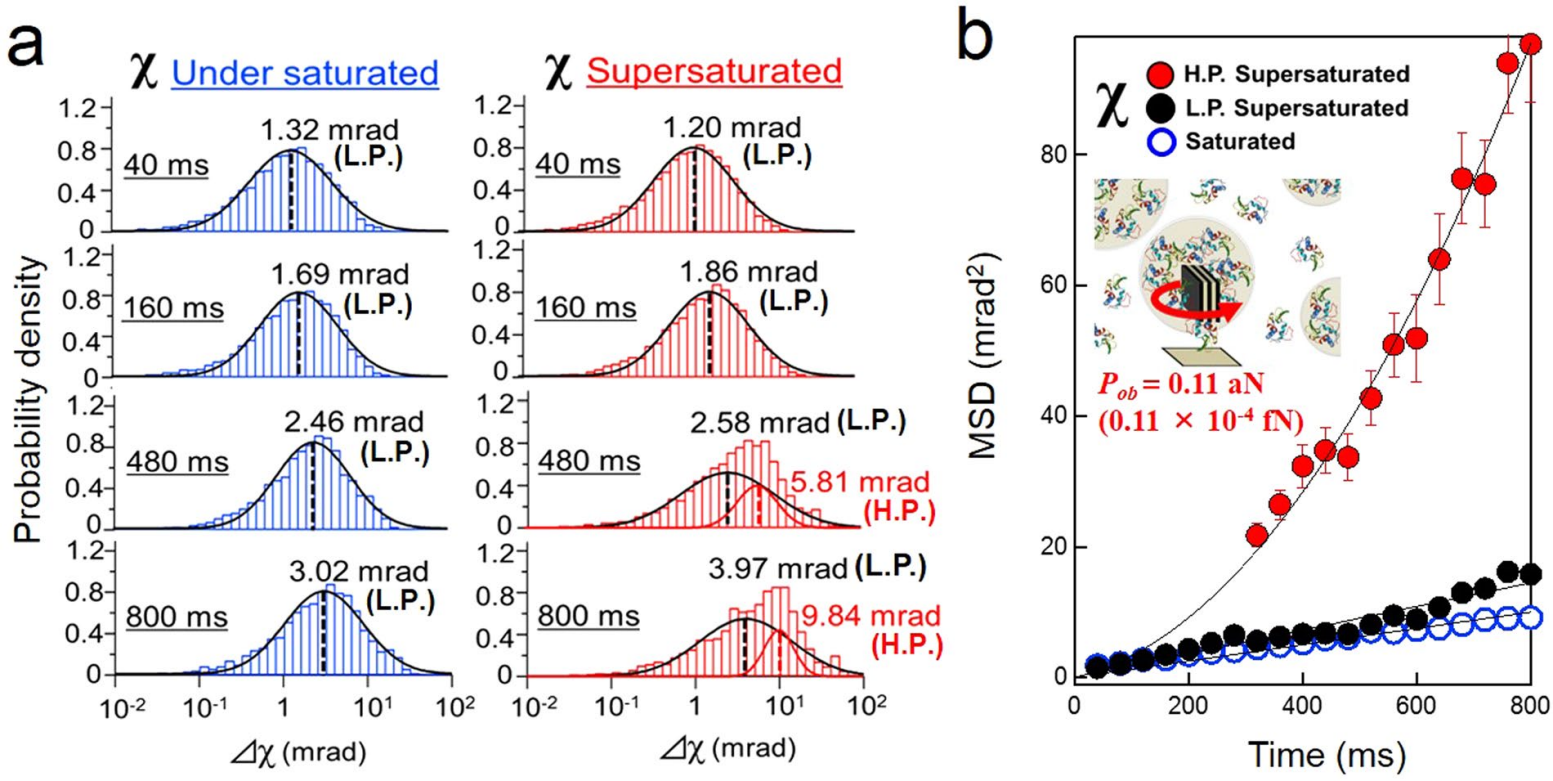
Gaussian distributions and MSD curves obtained from rotational displacement of gold nanocrystals on substrate, (**a**,**b**) Schematic drawing of a model of gold nanocrystal dispersed in the supersaturated lysozyme solution. (**c**) The distribution of the rotational angles in χ under saturated conditions was fitted by a single log-normal distribution. By contrast, the distribution of the rotational angles under supersaturated conditions was fitted by a double log-normal distribution between 320 and 800 ms. (**d**) MSD curves were calculated by the square of the peak position from the log-normal distribution fitting in the χ direction. The low-displacement peak was fitted with the equation *MSD* = *4Dt*, whereas the high-displacement peak was fitted with the equation *MSD* = *4Dt* + *(vt)* ^2^. Each H.P. was fitted using the plots ranging from 320 to 800 ms.

To perform a quantitative assessment, we calculated the log-normal distribution of the rotational angle displacements in the χ direction as a function of time, as shown in Fig. 2a. Each log-normal distribution was fitted by a single Gaussian distribution. We also performed a chi-squared test on each distribution. Chi-squared values are fitting parameters used as an index to determine to how similar a distribution is to a single Gaussian distribution. The chi-squared values are presented in Supplementary Table S1. The chi-squared statistical analysis revealed that gold nanocrystal displacement under supersaturated conditions in the χ direction increased from 40 ms (0.05) to 800 ms (0.23). Next, we used a double-Gaussian fitting for the displacement of the gold nanocrystals under supersaturated conditions in the χ direction. This calculation showed two distinct, separate distributions in different peak positions: a high-displacement peak (H.P.) and a low-displacement peak (L.P.), as shown in Fig. 2b. The MSD for each peak position is shown in Fig. 2b. The fitting parameters for each rotational direction are provided in Table S2. The MSD curve for normal diffusion shows characteristics highly similar to those of the L.P. MSD curve for gold nanocrystals under saturated conditions throughout the entire time range. By contrast, the MSD curve for H.P. shows a parabolic increase. Each MSD was fitted by an adequate MSD equation: by *MSD* = 4*Dt* for normal diffusion and by *MSD* = *4Dt* + *(Vt)* ^2^ for parabolic diffusion, where *D* is the diffusion constant [mrad^2^/ms] and *V* is the velocity [mrad/ms] of a gold nanocrystal. All the fitting parameters are provided in Table S2a–c. As previously reported^14,19^, the velocity (*V*) was used to calculate the anisotropic force field pressure (*P*_*ob*_), which was found to be 0.11 aN by taking the distance between the substrate and the centre of the gold nanocrystal to be 53.0 nm and the viscosity of the lysozyme solution to be 1.28 · 10^−3^ Pa · s (Figure S6a). This phenomenon was attributed to the displacement of the gold nanocrystals, reflecting the fluctuation of the networks coexisting under supersaturated conditions, as we reported previously^14^. Novel observations of lysozyme cluster properties have been reported in several recent studies^8,10,20^. In recent DLS studies of lysozyme clusters^10,12^, it was reported that if a cluster grew in a supersaturated solution, then the cluster’s surface free energy would be minimised because of Ostwald ripening^12^, and a size limit would be reached. As a result, the cluster growth process required several hours before the maximum size was reached. During this process, the cluster behaved as if it was in equilibrium with the solution and as if the cluster surface constantly exchanged protein molecules. Based on this reasoning, we calculated the received force *P*_*ob*_ (N) of the gold nanocrystals from the dynamic properties of the surrounding solution^14,19^. The *P*_*ob*_ value estimated from the DXT results precisely reflected the kinetic parameters of the cluster rearrangement process under supersaturated conditions.

**Figure 3:**
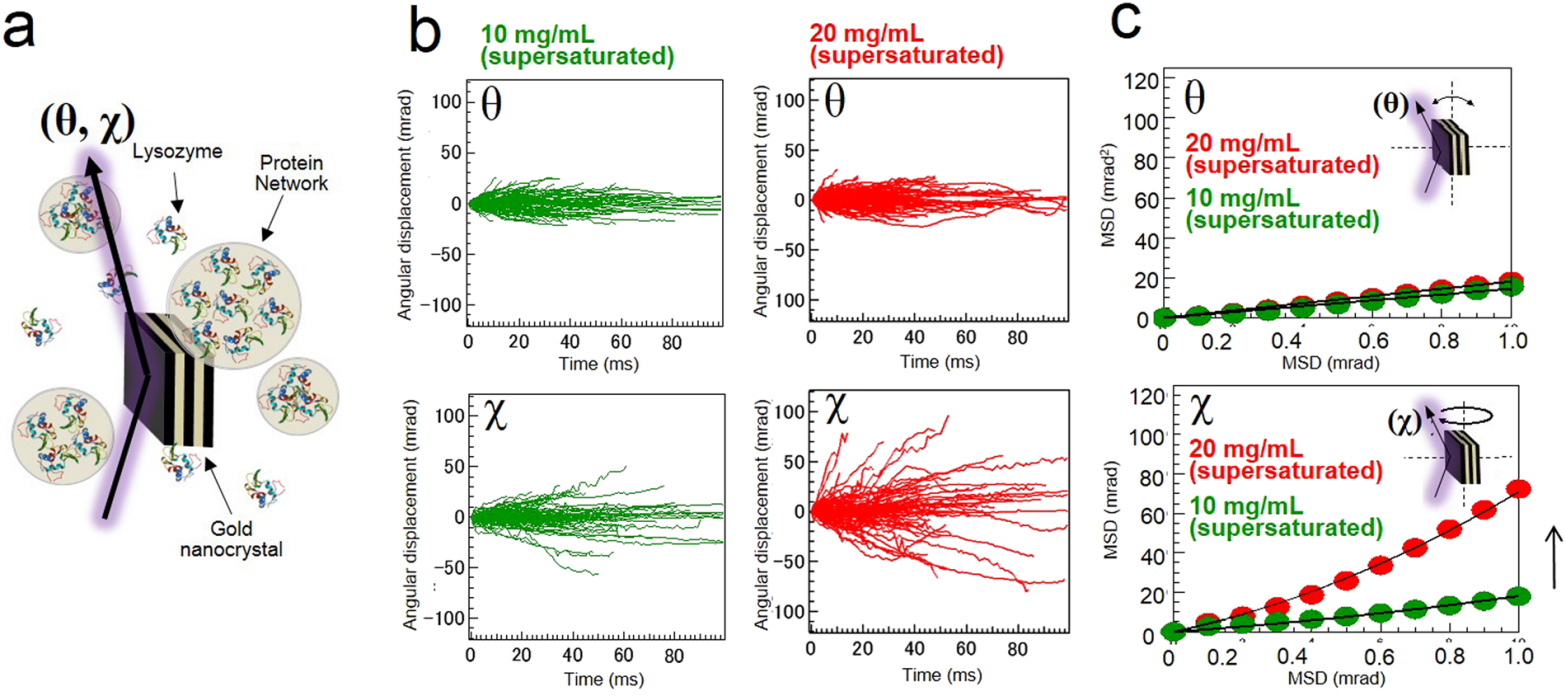
Statistical analysis of the rotational displacement of free-standing gold nanocrystals under supersaturated conditions. (**a**) Rotational angular displacement (θ and χ) of free-standing gold nanocrystals in 10 and 20 mg/mL lysozyme. (**b**) Average MSD curves for each sample condition.

**Figure 4:**
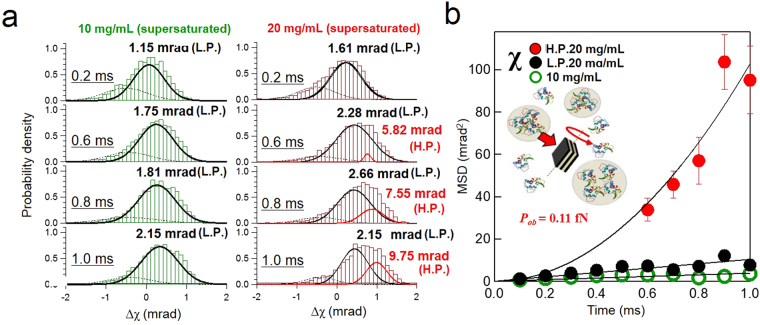
Gaussian distributions and MSD curves obtained from rotational displacement under free-standing gold nanocrystal conditions. (**a**) Distribution of rotational angles in the χ direction. The measurements in the 20 mg/mL solution were fitted with a double log-normal distribution. (**b**) MSD curves were calculated with the square of peak position from the log-normal distribution fitting in the χ direction. Analysis of the gold nanocrystal dynamics: MSD in the χ direction of the low displacement peak in each solution was fitted with the equation *MSD* = *4Dt*, whereas the high-displacement peak (H.P.) was fitted with the equation *MSD* = *4Dt* + *(vt)* ^2^, where *D* is a diffusion constant (rad^2^/s), *v* is velocity, and *t* is the measurement time. Each of H.P. was fitted using the plots ranging from 0.6–1.0 ms.

To confirm the repeatability of this significant characteristic of supersaturated solutions in excluding the effect of the adsorption conditions, we also performed free-standing DXT measurements under supersaturated lysozyme conditions (10–20 mg/mL lysozyme in 0.2 M sodium acetate with 3% w/v NaCl) to detect freely dispersed gold nanocrystals in solution. This experiment observes the dynamic properties of protein networks in bulk systems by extracting the distinctive fluctuations of gold nanocrystals. We compared the results of this experiment to our previous DXT results for the displacement in the χ direction using an inorganic supersaturated solution (−100 mrad/100 µs), and the gold nanoparticles moved more slowly than we expected (−75 mrad/10 ms), demonstrating that the viscosity of the supersaturated protein solution is three times lower than that of an inorganic supersaturated solution. Protein adsorption on the surface of the gold nanocrystals increased the hydrodynamic radii. Figure 3a,b shows the direction of X-ray exposure of the gold nanocrystals and the rotational directions of the θ and χ axes. Figures 3c,d and 4a,b show analysis results similar to those of the previous experiment. Figure 3c,d shows the rotational displacement of the gold nanocrystals in the θ and χ directions and the MSD curves of 10 and 20 mg/mL supersaturated lysozyme solutions. The results indicate that 20 mg/mL lysozyme showed more rapid dynamics than does 10 mg/mL lysozyme in the χ direction. As shown in Fig. 4a, the DXT measurements for 20 mg/mL lysozyme were confirmed to be clearly separated into two log-normal distributions. In contrast, the DXT for 10 mg/mL lysozyme converged to a single distribution. However, the distribution deviated only slightly from the single log-normal distribution near the 10^1^ mrad region in the χ direction (Fig. 4a). Next, the force field value that we calculated from the experiments was determined to be 0.11 fN using the same equation and known parameters, including the size of a gold nanocrystal and the viscosity of the supersaturated solution (1.28 · 10^−3^ Pa · s) at 25 °C (Figure S6b). Table S3 shows the gold nanocrystal sizes, rotational torque, and force field values from on-substrate and free-standing experiments. The experimental results also support the existence of two different dynamic modes (H.P. and L.P.) in the MSD analysis under the supersaturated conditions we used in previous experiments^14^. From these experiments, we concluded that crystal precursor solution states in both inorganic and protein solutions have nanoscale force fields. This observation is considered fundamental to the dynamics of molecular networks during the crystal nucleation process. For additional discussion, we also examined the differences between force field values and latent heat energy for each sample condition considering the reported network sizes of supersaturated inorganic^8,21,22^ and protein solutions ^12,13,23^ (Table S4). This analysis is considered as a clue to reveal a heat storage mechanism in supersaturated solutions.

In addition, we examined the circular dichroism (CD) spectra for the detailed internal structures of lysozyme, such as changes in secondary structure (200–250 nm) and aromatic amino acid conformation (275–300 nm), as shown in Figure S3. However, we observed only a small difference between supersaturated conditions with (4 °C) and without (25 °C) crystal generation.

In this study, we used DXT measurements to demonstrate that the rearrangement behaviours of lysozyme networks or clusters generated a force field *P*_*ob*_ that affected gold nanocrystals because of density and concentration fluctuations in the supersaturated solution. This force field value *P*_*ob*_ was found to be 11 ± 10^−4^ fN (0.11 aN) for fixed gold nanocrystals and 0.11 fN for free-standing gold nanocrystals. In our previous study, our group observed that the *P*_*ob*_ of an inorganic supersaturated solution (concentration: 6.4 M, viscosity: 3.18 · 10^−3^ Pa · s) generates a 1.60 fN force field to effect motion in free-standing gold nanocrystals in a bulk system^14^. This force field observed in the rotational direction is a common feature of nanoscale solute behaviour and may be an important aspect of the nucleation process in a supersaturated solution. Furthermore, force field parameters have been associated with the nucleation processes induced by shear flow^24,25^ and nanoscale viscous flow diffusion^21^ in supersaturated solutions. The discussion of supersaturated solutions and nanofluidics^22,23^ has the potential to reveal innovative approaches for understanding the crystal nucleation and growth mechanisms. Time-resolved analysis could then assign a key role to characterising the behaviour of solutes under supersaturated conditions.

We believe that this development will play a significant role in the study of other dilute supersaturated solutions, insoluble supersaturated solutions and ionic liquids by revealing the local dynamic structure of a variety of solutions. To this end, we are focusing on the modification of gold nanocrystals to develop the affinity for each sample, control the monodispersed gold nanocrystal size, and observe the transitional dynamics of gold nanocrystals.

## Method

### Diffracted X-ray tracking (DXT)

Substrates were fixed on gold nanocrystals using polyimide film (Toray Kapton) with a surface-deposited thin gold layer with an area of 250 Å (1 cm × 1 cm). To the substrate, 60 µL of 5 mg/mL lysozyme in 0.05 M phosphate buffer (pH 7.0) was added, and the solution was incubated at 4 °C for 6 hr. Then, 10 pM gold nanocrystal solution (60 µL) in 0.05 M phosphate buffer with 10 mM n-dodecyl-β-D-maltoside was incubated on the lysozyme-fixed substrate for 4 hr.

The saturated lysozyme solution (5 mg/mL) was prepared by dissolving 5 mg of lysozyme (Wako, Tokyo, Japan) in 1.0 mL of 0.2 M sodium acetate buffer with 3% w/v NaCl (pH 4.7). The solution was filtered through a 0.22 µm pore filter (Millipore, SLGV004SL Millex) to remove undissolved protein and other impurities. The supersaturated lysozyme solution was prepared by dispensing 5 µL of the saturated lysozyme solution onto the substrate on which the gold nanocrystals were fixed. Water evaporation was used to prepare the supersaturated lysozyme solution from the saturated solution by incubating 5 µL of the saturated solution under a dry atmosphere in a box with silica gel for 30 minutes. The final concentration of the supersaturated lysozyme solution was determined by the absorption coefficient at 280 nm, which was 2.64 (mL mg cm^−1^). A quartz cell with a path length of 14 µm (20/O/Q, Starna) was used for the absorbance measurements. Figure S1 shows the absorbance spectra of the saturated and supersaturated solutions. Based on the absorbance value at 280 nm, the lysozyme concentrations of the saturated and supersaturated solutions were determined to be 5 and 16 mg/mL, respectively. The weight reduction caused by water evaporation was 3.45 ± 0.15 mg for the 5 µL solution. The concentration of the supersaturated solution increased approximately three times from the concentration of the saturated solution. It was clear from the absorbance and weight loss measurements that the supersaturated concentration was higher than the tetragonal lysozyme crystal solubility at 25 °C based on the final concentrations of lysozyme and NaCl^26–28^. Crystallisation from the supersaturated condition was observed by incubating the supersaturated solution at 25 °C for 10 hours. The incubation cell was kept sealed throughout the experiment to avoid evaporation. The crystals obtained from the crystallisation experiment had a “sea urchin” crystal morphology^29,30^, as shown in Figure S2.

To perform free-standing DXT experiments, we prepared 20 and 10 mg lysozyme solutions at 0.2 M sodium acetate buffer (pH 4.7). Gold nanocrystals were dissolved by detaching the gold nanocrystals from 3 NaCl substrates in 1.0 mL of each solution. The NaCl concentration was confirmed by an osmometer (5600, IPROS) and varied from 3 to 4 w/v NaCl in each buffer.

Gold nanocrystals were fabricated by epitaxial growth on cleaved NaCl (100) (area: 7 mm × 7 mm) under a 10^−4^ Pa vacuum. Using atomic force microscopy (AFM), 1000 gold nanocrystals were observed on 100 μm^2^ NaCl substrates (Figure S5). The DXT measurement solution volume (for both saturated and supersaturated solutions) was 40 μL in a sample holder with a 50 μm X-ray transmission thickness. The experiment was performed at SPring-8 BL40XU (Hyogo, Japan). The DXT measurement layout was arranged as previously described^14^ (Figure S4). Fast DXT experiments (gold nanocrystal freely moving) were performed using X-rays with a total flux of 10^13^ photons/sec/0.1% bw with energy widths of 14.0–16.5 keV (undulator gap = 30.1 mm). Normal DXT experiments (gold nanocrystal fixed on substrate) used an X-ray flux reduced to one-thirtieth of the abovementioned value using a rotary disc shutter. The X-ray beam size was adjusted to 50 μm (vertical) by 50 μm (horizontal) using a four-quadrant slit and pinhole. The diffraction spot from a gold nanocrystal was detected when the lattice planes of the gold nanocrystal satisfied Bragg’s law (2*d* sin*θ* = *n λ*, where *d* is the interplanar spacing, *θ* is the angle between the incident beam and the relevant crystal planes, *n* is an integer, and *λ* is the wavelength of the incident beam). The X-ray diffraction spots from each nanocrystal were converted to visible light with an X-ray image intensifier (150 mm in diameter, V5445P, Hamamatsu Photonics, Japan), and time-resolved observation of the fast and normal DXT experiments was performed using a fast CMOS camera (768 pixel × 768 pixel, 25 µs/frame, 10 ms X-ray exposure time, SA 1.1, Photoron, Japan) and a CMOS camera (960 pixel × 720 pixel, 40 ms/frame, 3.6 ms X-ray exposure time, ORCA Flash 2.8, Photoron, Japan), respectively. DXT measurements were performed at room temperature (25 °C). The distance from the sample to the detector was approximately 100 mm.

Custom software written for IGOR Pro (Wavemetrics, Lake Oswego, OR) was used to analyse the diffraction spot tracks and trajectories. The time series of the angular position of the gold nanocrystals in samples in the θ and χ directions were smoothed with a 3-point moving average filter to reduce high-frequency noise. The intensity of the diffraction spots from the gold nanocrystals varied depending on the angular position of the diffraction spot in the θ direction and the corresponding flux of incident X-rays; therefore, the intensity of the diffraction spot was normalised by considering the X-ray background of the sample.

### Circular dichroism (CD) measurement

Solutions of 20, 12 and 14 mg/mL lysozyme were prepared in 0.2 M sodium acetate buffer with 3% w/v NaCl (pH 4.7), 0.1 M Tris-HCl buffer (pH 7.0) with 4 w/v NaCl, and 50 mM phosphate buffer (pH 4.7) with 4 w/v NaCl, respectively. The dependence of the CD and UV spectra of each solution on temperature is shown in Figure S3a–f. The resulting solution was shaken vigorously until all the lysozyme powder was isotropically dispersed. To dissolve the lysozyme completely, the solution was incubated at 50 °C for 5 minutes. When the solution appeared transparent, the solution was filtered through a 0.22 µm pore filter to remove undissolved or aggregated lysozyme and other impurities. The two sample cells used were made of quartz and featured path lengths of 14 and 140 µm. After injecting the sample into the quartz cell, parafilm was placed on the cell as a lid to avoid water evaporation during the measurement. The samples were analysed by continuous measurement at different temperatures (4, 10, 15, 20, and 25 °C). CD measurement was performed under a nitrogen atmosphere to prevent condensation on the cell surface. The measurement temperature (4, 10, 15, 20, 25 °C) was adjusted with a cooling machine circulating a water/methanol mixture solution. CD readings were performed using a JASCO J-815 circular dichroism machine.

## Electronic supplementary material

Supplementary figures and tables
