# Supplementary material for: Nanoscale Dynamics of Protein Assembly Networks in Supersaturated Solutions
Source: Sci Rep. 2017 Nov 1;7:13883. doi: 10.1038/s41598-017-14022-7 (PMC5665898; doi:10.1038/s41598-017-14022-7)
Supplement: Supplementary file 1 — Supplementary figures and tables [file 41598_2017_14022_MOESM1_ESM.pdf]

Nanoscale Dynamics of Protein Assembly Networks in Supersaturated Solutions.

Y. Matsushita[1], H. Sekiguchi[2], C. Jae Wong[1,7], M. Nishijima[3], K. Ikezaki[1,7], D. Hamada[4,5], Y. Goto[6], Y. C. Sasaki[1,2,7]

Corresponding author: Y. C. Sasaki
e-mail: ycsasaki@k.u-tokyo.ac.jp

1 . Graduate School of Frontier Sciences, The University of Tokyo, 5-1-5 Kashiwanoha, Kashiwa, Chiba, JAPAN
2 . Japan Synchrotron Radiation Research Institute, SPring-8, 1-1-1 Kouto, Sayo, Hyogo, JAPAN
3 Office for University - Industry Collaboration, Osaka University, 2-8, Yamadaoka, Suita, Osaka, JAPAN
4 Graduate School of Engineering, Kobe University, 7-1-48 Minato-jima, Minami, Kobe, Hyogo, JAPAN
5 SPring-8 / RIKEN, 1-1-1 Kouto, Sayo, Hyogo, JAPAN
6 Institute for Protein Research, Osaka University, 3-2 Yamadaoka, Suita, Osaka, JAPAN
7 AIST-UTokyo Advanced Operando-Measurement Technology Open Innovation Laboratory (OPERANDO-OIL), National Institute of Advanced Industrial Science and Technology (AIST), Chiba 277-8568, Japan

Supplementary Information

-Supplementary Figure

-Supplementary Table

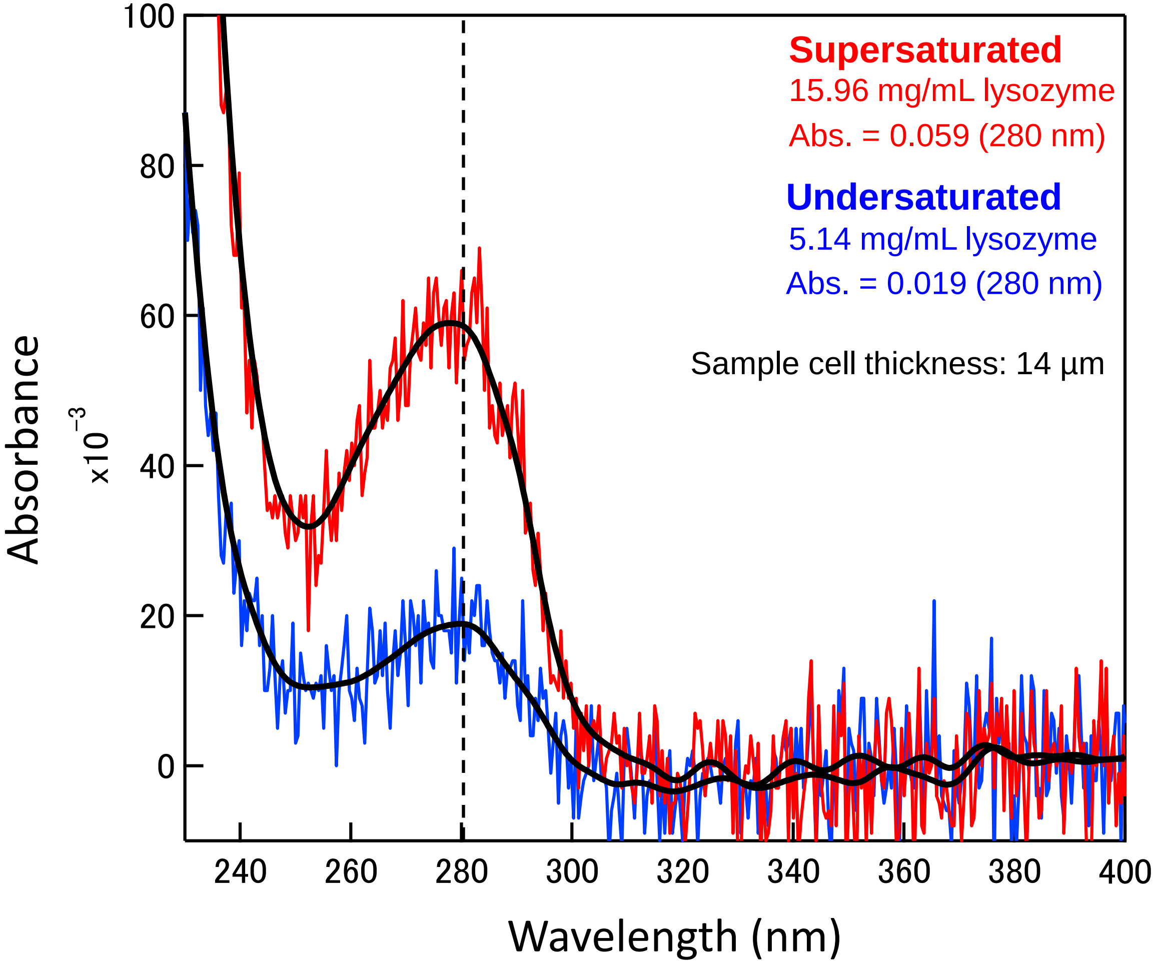

Figure S1 | UV-absorbance spectra of the undersaturated (5 mg/mL) lysozyme solution in 0.2 M sodium acetate buffer with 3 w/v % NaCl (pH 4.7) and the supersaturated solution prepared by evaporating water from the undersaturated solution for 30 min under dry air conditions. Lysozyme concentration of saturated and supersaturated solutions were approximately 5 mg and 16 mg/mL respectively. The concentration was calculated based on the Lambert-Beer equation. The sample cell thickness and absorbance coefficient ε used were 14 µm and 2.64 (mL・mg cm^{-1}), respectively.

Figure S1 (Y. Matsushita)

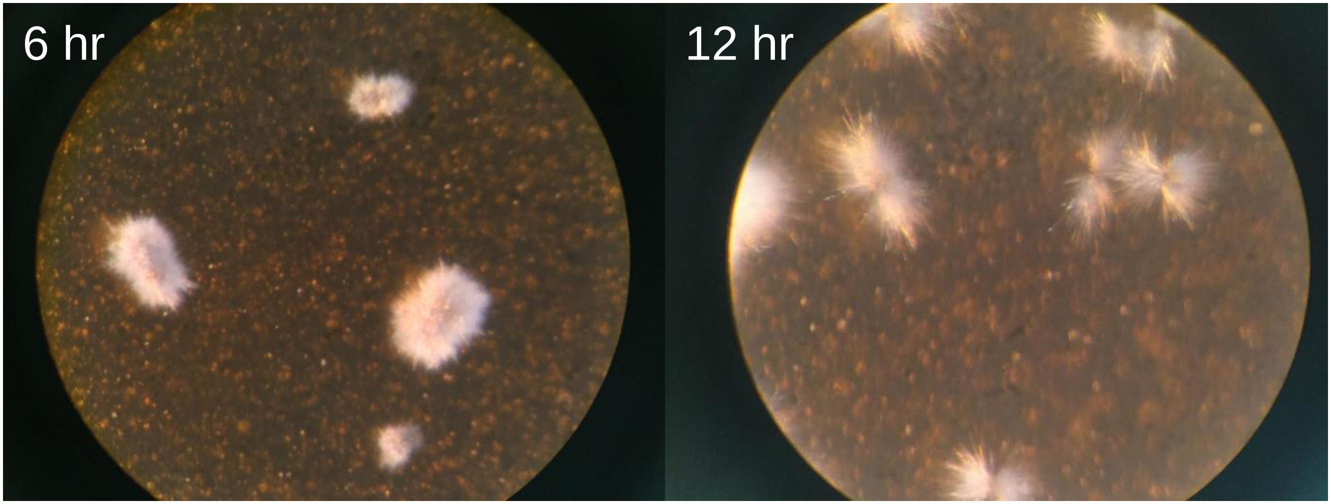

Figure S2 | Crystal from 1 mL of supersaturated lysozyme solution incubated in sealed cell for 12 hr at 25°C. The image on the left is a microscopy image of generated crystals at 6 hr incubation, and the image on the right side shows the crystals at 12 hr incubation. The crystals appear sea-urchin-shaped.

Figure S2 (Y. Matsushita)

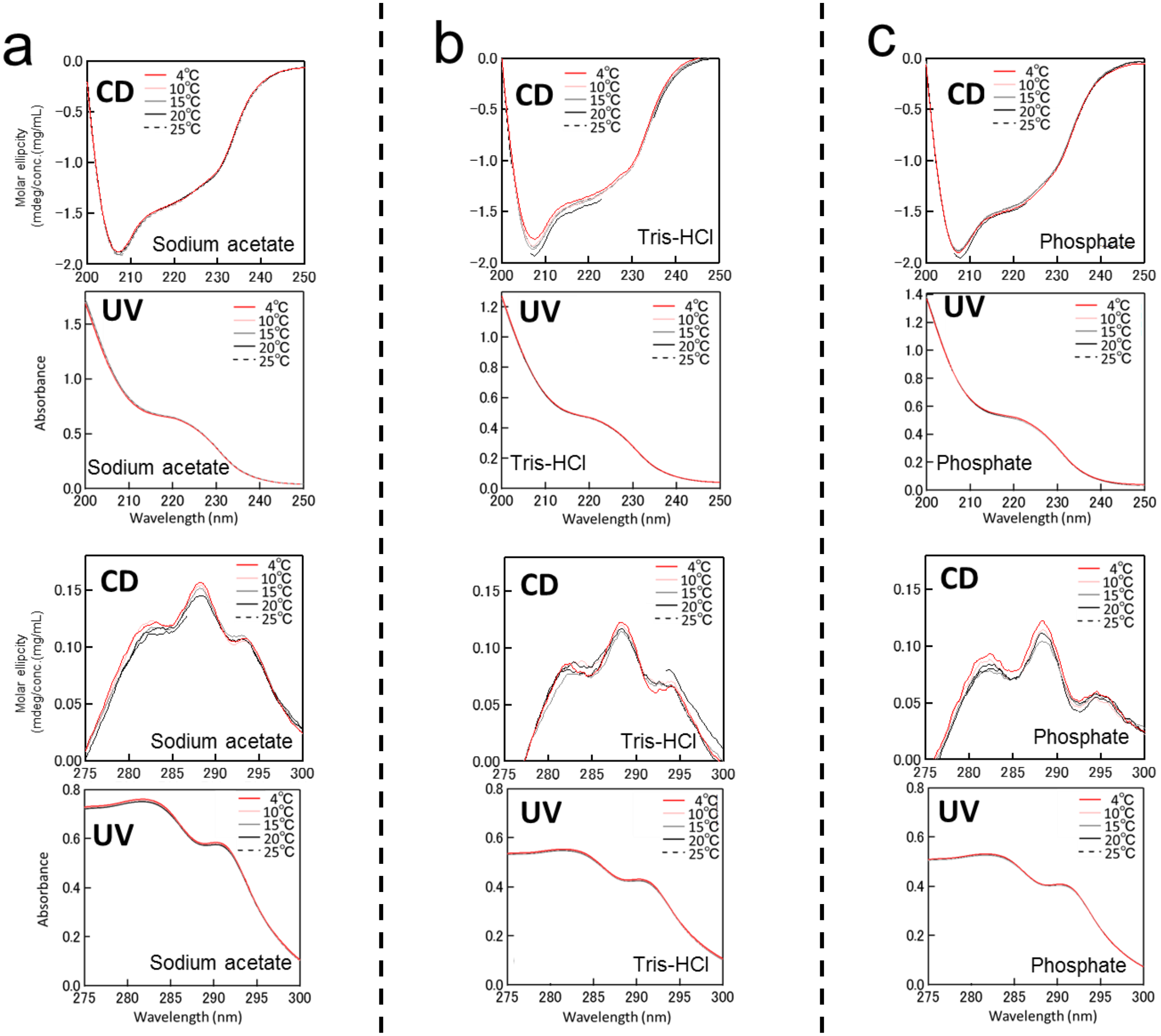

Figure S3 | CD and UV-absorbance spectra for the lysozyme in sodium acetate, Tri-HCl and phosphate buffer with NaCl at 25, 20, 15, 10, and 4°C. **a.** CD and UV-absorbance spectra of 20 mg/mL lysozyme solution at 0.2 M sodium acetate with 3 % w/v NaCl (pH 4.7) **b.** CD and UV-absorbance spectra of 12 mg/mL lysozyme solution at 0.1 M Tris-HCl with 4% w/v NaCl (pH 7.0) **c.** CD and UV-absorbance spectra of 14 mg/mL lysozyme solution at 0.05 M phosphate buffer with 4% w/v NaCl (pH 7.0).

Figure S3 a-c (Y. Matsushita)

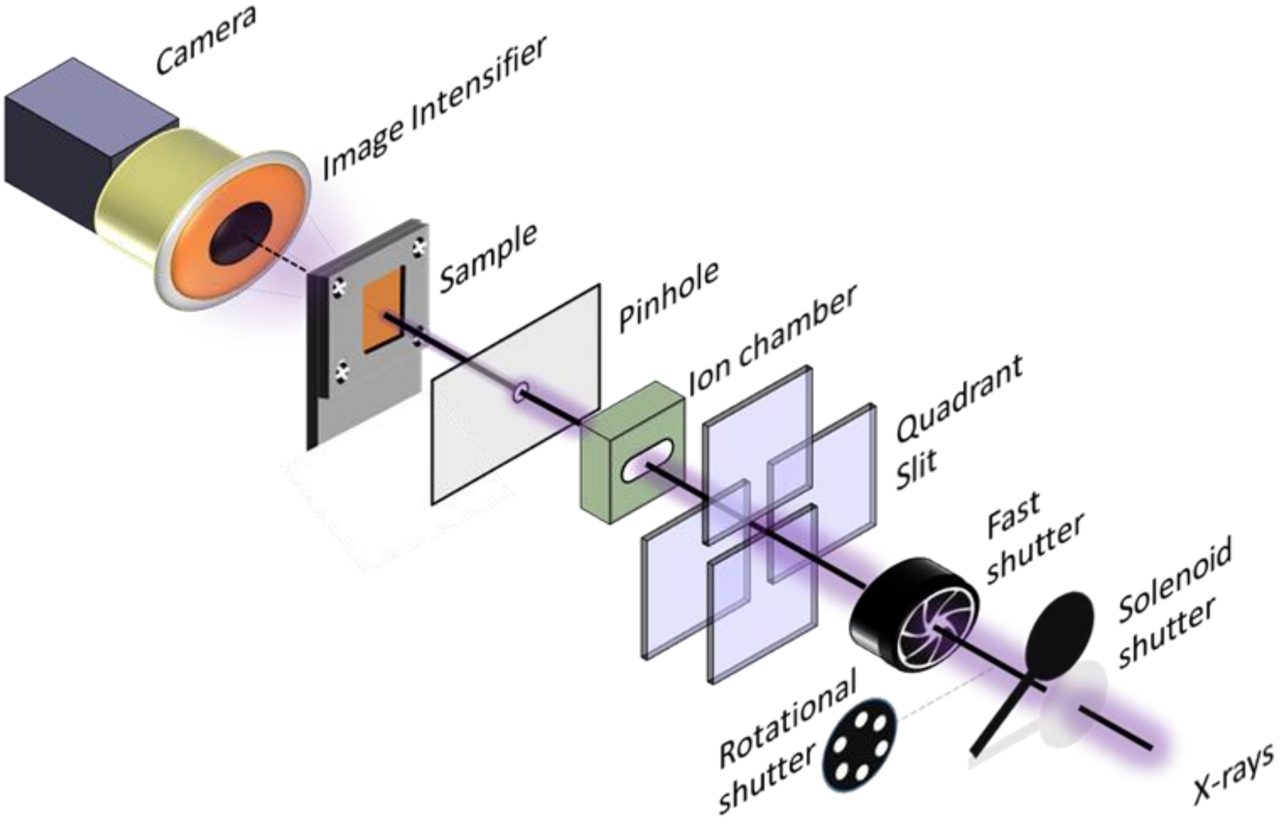

Figure S4 (Y. Matsushita)

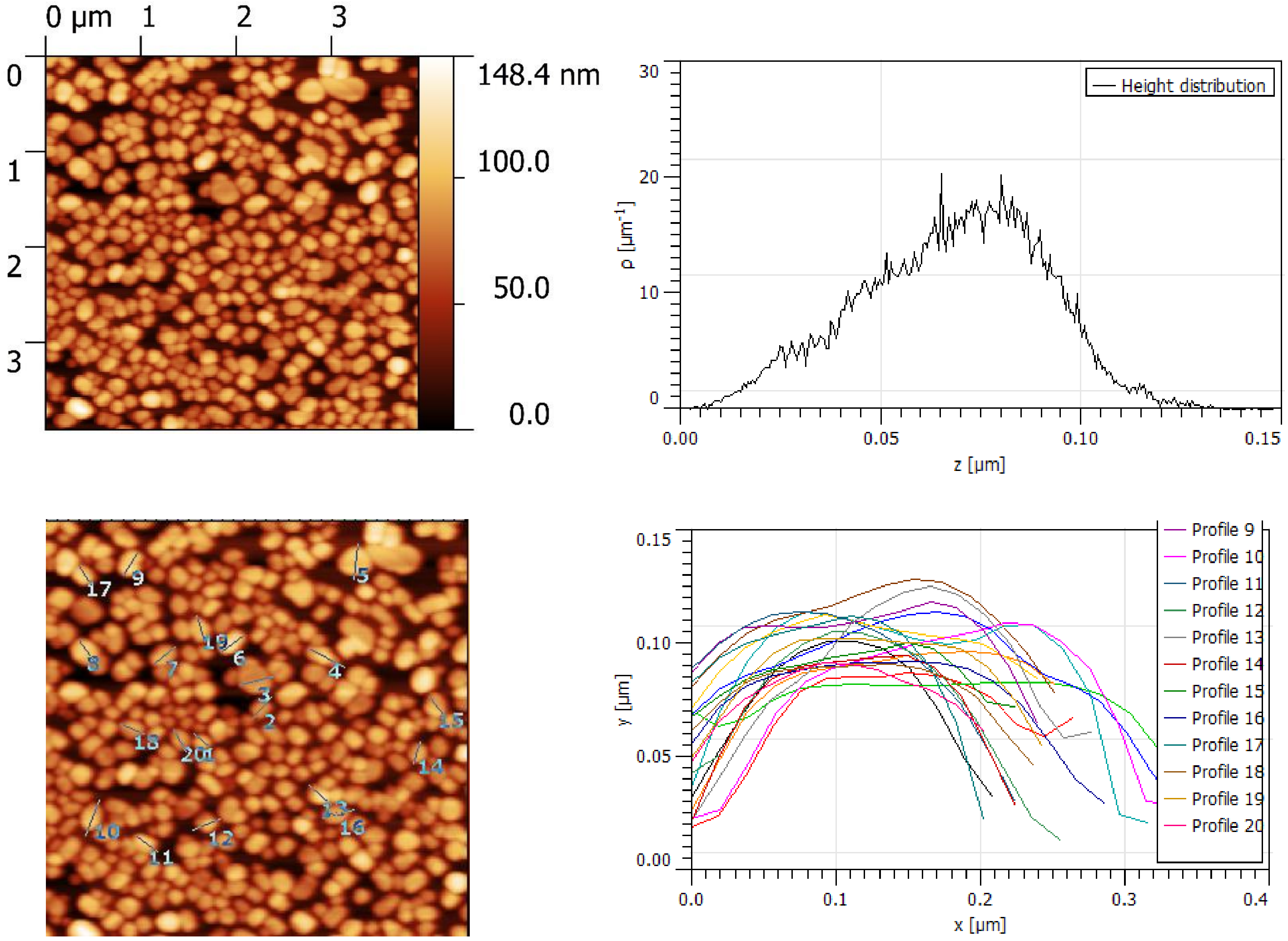

Figure S5 (Y. Matsushita)

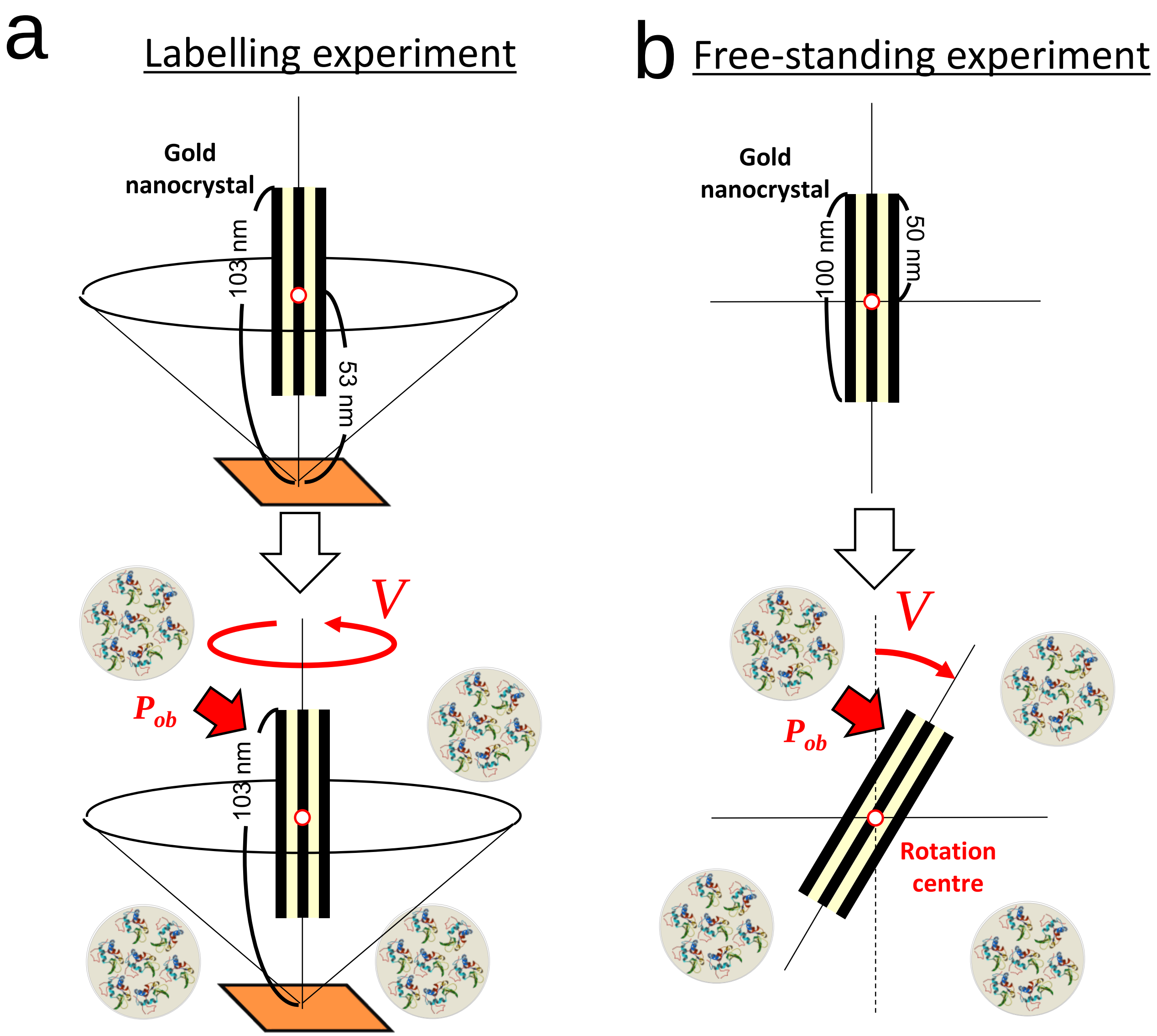

$$P_{ob} = \Gamma / h = 2\pi C_{rd} V / 2\pi h$$

$$C_{rd} = \pi\eta h^3$$

P_{ob}: Observed force pressure
Γ: Rotational Torque
V: Rotational velocity
h: Radius of a gold nanocrystal
η: Viscosity of sodium acetate
C_{rd} : Observed force pressure

Figure S6 a,b (Y. Matsushita)

Chi Square χ^2

	θ		χ	
	Under saturated	Supersaturated	Under saturated	Supersaturated
40 ms	0.106275	0.118303	0.0492272	0.0514004
80 ms	0.0958908	0.0975653	0.0521556	0.0474151
120 ms	0.103876	0.0795117	0.0711884	0.0489464
160 ms	0.104749	0.0898268	0.0667383	0.0696665
200 ms	0.0754286	0.101408	0.0798393	0.0844019
240 ms	0.0669029	0.0921008	0.0909116	0.0723509
280 ms	0.0656418	0.088687	0.0727323	0.0867266
320 ms	0.0791977	0.102017	0.0666153	0.0824056
360 ms	0.0621676	0.11248	0.0668326	0.0897144
400 ms	0.0547291	0.108787	0.0701992	0.0963251
440 ms	0.0436539	0.108223	0.0867197	0.124518
480 ms	0.0686354	0.12511	0.090366	0.117614
520 ms	0.0797699	0.135603	0.129437	0.134629
560 ms	0.0775212	0.132506	0.0773999	0.14003
600 ms	0.144394	0.144394	0.112953	0.141262
640 ms	0.0491626	0.14473	0.0785434	0.151004
680 ms	0.0588238	0.134082	0.081689	0.177628
720 ms	0.053287	0.187748	0.0777182	0.213184
760 ms	0.0490356	0.188085	0.0640831	0.202903
800 ms	0.0497504	0.184193	0.0825997	0.23451

Table S1 | Chi-squared χ^2 values obtained from Gaussian fitting in χ and θ directions of gold nanocrystals fixed on substrate in undersaturated and supersaturated conditions.

Table S1 (Y. Matsushita)

a

χ 40 – 800 ms	*D [mrad^2/ms]*	*V [mrad/ms]*
Under saturated	0.0031±8e^{-5}	-
L.P. Supersaturated	0.0045±1e^{-4}	-
H.P. Supersaturated	0.0051±2e^{-3}	0.011±6e^{-4}

b

χ 0.1 – 1.0 ms	*D [mrad^2/ms]*	*V [mrad/ms]*
10 mg/mL	1.24 ± 0.05	-
L.P. 20 mg/mL	2.59 ± 0.22	-
H.P. 20 mg/mL	0.00 ± 12.30	10.13 ± 2.82

Table S2 | Diffusion constant D and velocity V obtained by fitting of MSD curves in Figures 2b and 4b. a, Fitting parameters of gold nanocrystals on substrate in undersaturated and supersaturated conditions (Figure 2b). **b,** Fitting parameters of freely moving gold nanocrystals under 10 and 20 mg/mL lysozyme conditions (Figure 4b).

Table S2 a, b (Y. Matsushita)

	On substrate	Free-standing
Gold nanocrystal size (nm)	53	50
Torque (fN・nm)	5.49×10^{-3}	5.65
Force field (fN)	0.106 x 10−3	0.108

Table S3 (Y. Matsushita)

Table S3 | Comparison of inorganic and protein supersaturated solutions. "Inorganic" and "protein" refer to sodium acetate and lysozyme molecules, respectively. Concentration, diffusion constant, velocity and observed force values were obtained from free-standing DXT measurements as indicated in the table. The values in parentheses correspond to known values (molecular size, molecular weight, viscosity[15]) from previous studies.

DXT	Inorganic	Protein
**Concentration**	6.4 M	$2.1 * 10^{-3}$ M
**Molecular Size, weight**	(0.6 nm, 136.08 g/mol)	(4 nm, 14307 g/mol)
**Viscosity**	(3.18 kg/m s)	(1.28 kg/m・s)
**Crystal Latent Heat of Fusion**	14 kJ/L (Trihydrate)	0.045 kJ/L (Tetragonal)
**Diffusion Constant**	0.30 $mrad^2$/ms	1.84 $mrad^2$/ms
**Velocity (Rotational)**	64 mrad/ms	10 mrad/ms
**Force Field**	1.60 fN	0.11 fN

Table S4 (Y. Matsushita)

Comparison of table of free-standing gold nanocrystal DXT results and the known parameters for inorganic and protein supersaturated solutions. As indicated, excess molecular dissolution conditions retain latent heat energy. The difference between inorganic and protein samples was approximately 300-fold; by contrast, the force-field values from DXT differed by approximately 15-fold, indicating that the conversion of force-field energy to latent heat energy is not as simple as we expected. The network sizes were considered as possible causes of this difference, because inorganic network sizes are estimated to range from 1 to 10 nm under supersaturated conditions, whereas protein networks have been reported at a scale of several tens to hundreds of nm. These differences led us to conclude that the force-field values of unit distances in each sample corresponded to a difference of several tens to several hundreds of times using a first-order approximation. Furthermore, these differences were similar to the 300-fold difference in the amount of latent heat in each sample. The results also indicated that a single unit of the inorganic network retained a larger amount of latent heat than a single unit of the protein network. Therefore, this force-field parameter is strongly related to molecular nanoscale behaviours, energy-saving mechanisms, and nucleation processes in supersaturated solution.
